# Re-Emergence of BTV-4 in Sheep Farms in Kosovo, 2020: A Retrospective Study

**DOI:** 10.1155/2023/3112126

**Published:** 2023-02-27

**Authors:** Seyma S. Celina, Simon King, Martin Ashby, Katie Harris, Noemi Polo, Mentor Alishani, Avni Robaj, Afrim Hamidi, Driton Sylejmani, Carrie Batten, Jiří Černý

**Affiliations:** ^1^Center for Infectious Animal Diseases, Faculty of Tropical AgriSciences, Czech University of Life Sciences Prague, Prague, Czech Republic; ^2^The Pirbright Institute, Ash Road, Pirbright, Woking, Surrey GU24 0NF, UK; ^3^Department of Veterinary Medicine, Faculty of Agriculture and Veterinary, University of Prishtina “Hasan Prishtina”, Prishtina, Kosovo

## Abstract

Kosovo has previously seen two bluetongue (BT) epizootics, each caused by a different serotype, BTV-9 in 2001 and BTV-4 in 2014. Since 2014, no clinical cases of BT have been reported in Kosovo. In September, 2020, clinical signs suggestive of BTV infection were observed in several sheep farms in Kosovo. Blood samples from sheep (*n* = 40) were collected and subjected to further molecular investigations. Molecular analyses confirmed BTV serotype 4 (BTV-4) infection in thirty-six sheep from five different farms across two different regions. Full genome sequence analyses indicated that the BTV-4 strains (KOS2020/01 and KOS2020/02) detected in Kosovo in 2020 had high sequence identity (99.9%-100%) with a strain responsible for an outbreak in North Macedonia in July, 2020, (MKD2020/06) and with previous isolates (≥99.3%) from Greece, Hungary, and France. The percent nucleotide sequence (nt%) identity and phylogenetic analyses suggest that the incursion of BTV-4 into Kosovo was a re-emergence of a previously seen strain and not a novel reassortant. This could be due to a reintroduction of the strain into the region or from subclinical circulation which had been ongoing and underreported for years. Surveillance across Kosovo and the Balkan region to monitor the circulation of BTV is crucial if outbreaks are to be brought under control.

## 1. Introduction

Bluetongue virus (BTV), a member of the *Orbivirus* genus (*Reoviridae* family) and the aetiologic agent of bluetongue (BT) disease, is a nonenveloped double-stranded RNA virus with 27 serotypes currently recognised. The viral genome consists of 10 segments encoding seven structural (VP1-VP7) and five nonstructural (NS1-NS5) proteins [1, 2]. The structural protein VP2, encoded by BTV segment 2, is the most variable structural protein and major determinant of serotype specificity [3].

Although vertical and horizontal transmissions have also been described, the transmission of BTV to ruminant hosts occurs through a blood-feeding activity of midges of the genus *Culicoides* (*Diptera* and *Ceratopogonidae*) [4, 5]. BT affects both domestic and wild ruminant species. Manifestation of BT varies depending on the serotype and species involved [6], ranging from the absence of clinical signs to mild fever, inflammation of the mucous membranes, congestion, swelling, haemorrhages, abortion, and death. Although all ruminants are susceptible to BT disease, sheep are generally the most affected species, with more severe clinical presentation [7]. Cattle and goats do not usually show any clinical signs of disease, allowing them to act as reservoir hosts and enabling further silent spread by *Culicoides* [8, 9]. Due to its potential for rapid spread causing significant production loss for the sheep and cattle industry, and its importance to the international trade in livestock, it has been classified as a notifiable disease by the World Organisation for Animal Health (WOAH). Surveillance and control of BTV are crucial in preventing spread of the disease which would otherwise lead to considerable economic impacts due to trade restrictions and production losses.

The distribution of the virus coincides with the geographic range of its vector species resulting in the presence of BTV on all continents except Antarctica [7]. Before 1998, several sporadic incursions of BTV from sub-Saharan Africa and the Middle East occurred in Spain and Portugal (BTV-10) [10, 11] and on Greek islands (BTV-4) [12]. Since 1998, the distribution of BTV in Europe has changed dramatically with several incursions by multiple serotypes (serotypes 1, 2, 3, 4, 6, 8, 9, 11, and 16) [13]. After the detection of BTV-9 on several Greek islands in 1998, this serotype gradually spread northwards and westwards through mainland Greece and beyond, eventually reaching Bulgaria, North Macedonia, Montenegro, and Bosnia and Herzegovina in 2001 [14]. In the summer of 2001, the serotype also spread to Kosovo, marking the first clinical BT cases to be seen in the country [15]. Serological surveys of cattle, sheep, and goats carried out in subsequent years suggested that BTV had circulated subclinically among the cattle and sheep populations of Kosovo from 2002 to 2004 [15]. Kosovo saw its second incursion of the disease in 2014 during the widespread circulation of BTV-4 throughout the Balkan Peninsula. Since 2014, no clinical cases of BT had been reported in Kosovo despite limited BTV outbreaks in the surrounding regions. However, in 2020, after the re-emergence of BTV-4 in the North Macedonia [16], clinical cases of BT were also observed in Kosovo.

In this study, we described the recent re-emergence of BTV-4 in Kosovo and utilised full genome sequencing to characterise the strain responsible for the outbreak.

## 2. Methods

### 2.1. Samples

**Figure 1:**
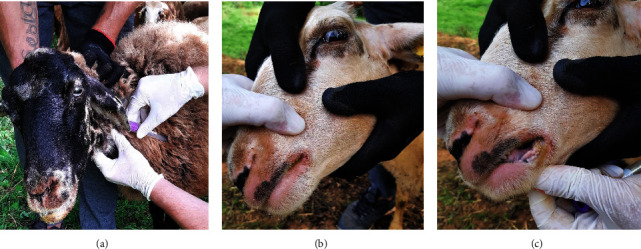
Clinical signs observed in sheep affected by BTV on farms in Kosovo. (a) Nasal discharge, erosion of the nasal planum, and excessive salivation. (b) Scabby mouth and abrasions on the bare skin around the lips. (c) Erosions and ulceration of oral mucosa. Lips are swollen with greyish brown necrotic deposition.

In September, 2020, clinical signs suggestive of BTV infection (facial oedema, fever, nasal discharge, anorexia, and respiratory problems) were observed in sheep (Figure 1) at three farms located in Prizren (southern Kosovo) and two farms located in Vushtrri (northern Kosovo). EDTA blood samples were collected from a total of 40 sheep (39 females and 1 male of varying ages) displaying clinical signs at these farms.

### 2.2. Initial Molecular Diagnosis

The EDTA blood samples were sent to the Centre for Infectious Animal Diseases, FTZ, in Prague and tested for BTV RNA using real-time RT-PCR [17]. The samples were subsequently submitted to the WOAH reference laboratory for BT at The Pirbright Institute, UK, for confirmatory diagnosis and further molecular investigations.

### 2.3. Virus Isolation

Cell culture supernatant from a previously passaged (KC1) sample from the 2014 outbreak in Kosovo (KOS2014/01) was further passaged once in KC cells. Cells were inoculated with 200 *µ*l of KOS2014/01 (KC1) supernatant diluted in 1.8 mL of fresh growth medium, consisting of 88% Schneiders Insect medium (Merck), 10% FBS (Gibco), 1% penicillin/streptomycin (100 U/mL and 100 *µ*g/mL) (Sigma-Aldrich), and 1% amphotericin B (2.5 *µ*g/mL) (Sigma-Aldrich). The KC cells were then incubated at room temperature for 30 mins, before an additional 8 mL of fresh media was added to further supplement the cells. Following 7 days incubation at 27°C, the cells were scraped into the media and centrifuged at 1,000 × *g* for 5 min. The supernatant was drawn off and the cell pellet was taken forward for extraction.

### 2.4. Nucleic Acid Extraction

Prior to real-time RT-PCR assays, total nucleic acid was extracted from 200 *µ*l of EDTA blood using the MagMAX Core Nucleic Acid purification kit (ThermoFisher Scientific) on a KingFisher Flex automated robot. Nucleic acid was eluted in 90 *µ*l of elution buffer.

Prior to next-generation sequencing, RNA was extracted using TRIzol LS and the MagMAX mirVana Total RNA isolation kit (ThermoFisher Scientific). Briefly, 250 *µ*l of EDTA blood was mixed with 750 *µ*l TRIzol LS and incubated for 5 min, followed by the addition of 200 *µ*l cold chloroform. For cell pellets from virus isolation, 2 mL of TRIzol was added to the pellet and incubated for 5 min, followed by the addition of 400 *µ*l cold chloroform. Samples were centrifuged at 12,000 × *g* for 15 min at 4°C and the resulting aqueous layer was drawn off. 300 *µ*l of aqueous phase was used as input for the MagMAX mirVana Total RNA isolation kit on a KingFisher Flex automated robot and RNA eluted in 55 *µ*l of elution buffer.

### 2.5. Real-Time RT-PCR

Extracted RNA was denatured at 95°C for 5 min prior to each real-time RT-PCR assay. All BTV real-time RT-PCR assays were performed on an Applied Biosystems 7500 Fast Real-Time PCR instrument using the Express One Step SuperScript qRT-PCR kit (ThermoFisher Scientific).

The Seg-10 real-time RT-PCR assay [18] was performed with modifications from the published conditions; the final 20 *µ*l reaction comprised 1 × reaction mix, 400 nM forward and reverse primers, 200 nM probe, 0.4 *µ*l ROX, 2 *µ*l enzyme, and 5 *µ*l of RNA. The cycling conditions for the Seg-10 assay were as follows: 50°C for 15 min, 95°C for 20 s, followed by 45 cycles of 95°C for 3 s, 56°C for 30 s, and 72°C for 30 s.

Each serotype-specific real-time RT-PCR [17] was performed in a final volume of 20 *µ*l comprising 1 × reaction mix, 400 nM forward and reverse primers, 100 nM probe, 0.4 *µ*l ROX, 2 *µ*l enzyme, and 3 *µ*l of RNA. Cycling conditions were as follows: reverse transcription at 50°C for 15 min and 95°C for 20 s, followed by 45 cycles of 95°C for 3 s and 60°C for 30 s.

### 2.6. cDNA Synthesis

Extracted RNA was denatured at 95°C for 5 min before 8 *µ*l was used as input into the SuperScript III First Strand Synthesis system (ThermoFisher Scientific) using random hexamers as a primer and following the manufacturer's protocol. First strand cDNA was converted into double-stranded cDNA using the NEBNext Ultra II Nondirectional RNA Second Strand Synthesis module (New England BioLabs), following the manufacturer's protocol but increasing the incubation time to 2.5 hours. cDNA reactions were purified using the Illustra GFX PCR DNA and Gel Band purification kit (Cytiva), according to manufacturer's instructions, and eluted in 30 *µ*l of elution buffer type 4.

### 2.7. Next-Generation Sequencing

For the sample from virus isolation, double-stranded cDNA was quantified using the Qubit dsDNA High Sensitivity kit and diluted to 0.2 ng/*µ*l for library preparation. Libraries were generated using the Nextera XT library preparation kit (Illumina), following the manufacturer's protocol.

Viral targeted enrichment was carried out using a custom myBaits panel (Daicel Arbor Biosciences) to sequence directly from clinical material. Barcoded libraries were prepared using the Illumina DNA Prep with Enrichment kit (Illumina) following the manufacturer's protocol, but using 11 cycles during the amplification of tagmented DNA. Barcoded libraries were enriched using a custom myBaits probe panel (Daicel Arbor Biosciences) designed against all BTV segments from all known serotypes. The high sensitivity protocol incorporating two rounds of probe hybridisation was followed, with a hybridisation temperature of 63°C. Size distribution of enriched libraries was determined using the D1000 kit on a TapeStation (Agilent).

All libraries underwent paired end sequencing (2 × 150 cycles) on an Illumina MiSeq using v2 reagents.

### 2.8. Genome Assembly

Consensus genome sequences were assembled using a custom pipeline. Reads were aligned against each segment of a reference genome (BTV-4 MKD2020/06; GenBank accessions MT879201-MT879210) using bwa-mem v0.7.12 and the bcftools package v1.3.1 to call the consensus sequence. Mapping statistics were generated with weeSAM v1.5.

### 2.9. Sequence and Phylogenetic Analysis

Consensus sequences for each segment were compared to publicly available sequences using BLAST. Pairwise alignments between closely related sequences were performed to calculate the percentage nucleotide identity.

Reference sequences for segments 2 and 6 encompassing each BTV serotype were downloaded from GenBank and trimmed to their coding sequence. Maximum likelihood phylogenetic trees were constructed in MEGA X using the Tamura-Nei model. Support was tested using 1,000 bootstrap replicates.

### 2.10. Ethical Statement

The work carried out in this study was a part of the Pirbright Institute's role as a WOAH reference laboratory for the diagnosis of BTV and therefore, no ethical approval was required.

## 3. Results

### 3.1. Real-Time RT-PCR Analysis

Of the 40 blood samples collected across five farms, 36 were positive for BTV by real-time RT-PCR with *C*_*T*_ values in the range of 18.7–26.9. Serotype-specific real-time RT-PCRs targeting BTV-4 and BTV-9 were performed for the determination of serotype as only these serotypes have previously circulated in the country. All BTV RNA-positive samples (*n* = 36) were positive using the BTV-4 serotype-specific real-time RT-PCR assay (*C*_*T*_ range 18.5–27.9). No amplification was observed for the serotype-specific real-time RT-PCR assay targeting BTV-9. Altogether, these results confirmed the clinical diagnosis and the re-emergence of BTV-4 in Kosovo.

### 3.2. Sequence and Phylogenetic Analysis

Two EDTA blood samples were chosen for full genome sequencing; KOS2020/01 from the Vushtrri municipality in northern Kosovo and KOS2020/02 from the Prizren municipality in southern Kosovo. A virus isolate (KOS2014/01) from the previous BTV-4 outbreak in Kosovo in 2014 was also sequenced for comparison to the present circulating strain. The genomes were sequenced with an average read depth of 18,930x; 20,605x; and 3,585x per base for KOS2020/01, KOS2020/02, and KOS2014/01, respectively. Full sequences were obtained for all segments of all three samples, except segment 1 of KOS2020/01 (2 bases missing from 3′ terminus) and segment 5 of KOS2020/01 (2 bases missing from 5′ terminus) and KOS2014/01 (3 bases missing from 5′ terminus). The sequences were deposited in GenBank with the following accession numbers: OP186396-OP186405 (KOS2020/01), OP186406-OP186415 (KOS2020/02), and OP186416-OP186425 (KOS2014/01).

**Figure 2:**
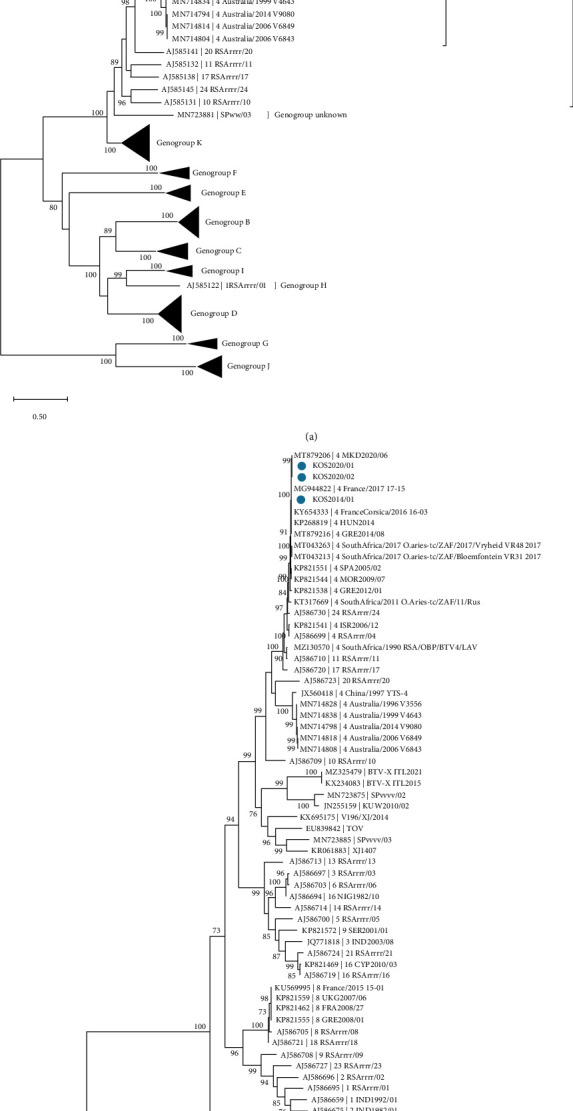
Phylogenetic relationship of BTV-4 from outbreaks in Kosovo in 2014 and 2020 based on (a) VP2 coding region and (b) VP5 coding region. Sequences produced in this study are labelled with a blue circle. Maximum likelihood trees (1,000 bootstrap replicates) were constructed using MEGA X software. Bootstrap support values of ≥70% are shown.

Across all ten segments, the two samples from 2020 shared >99.9% nucleotide identity to each other. Comparison of the nucleotide sequences of all ten segments of the 2020 samples (KOS2020/01 and KOS2020/02) using BLAST+ 2.13.0 [19] showed that they share high sequence identity with the recent BTV-4 strain circulating in North Macedonia in 2020 [16] (Table 1). The percent nucleotide sequence (nt%) identity of BTV segment 2, the major determinant of serotype specificity, against the available BTV-4 segment-2 sequences showed that both KOS2020/01 and KOS2020/02 were most closely related to MKD2020/06 (99.93%; 99.97%), GRE2014/08 (99.73%, 99.76%), BTV4-HUN2014 (99.66%, 99.69%), and KOS2014/01 (99.72%, 99.76%). Moreover, segments 3, 4, 5, 6, 7, 8, 9, and 10 (encoding VP3, VP4, NS1, VP5, VP7, NS2, VP6/NS4, and NS3/NS3a, respectively) of KOS2020/02 showed 100% identity with an isolate of BTV-4 from North Macedonia, 2020 (Strain ID: MKD2020/06) (Table 1). Indeed, all segments of the two samples share >99% nucleotide identity with the sequences of the BTV-4 strains that circulated during the previous Balkan BTV-4 epizootic and in Europe (Strain IDs: MKD2020/06 (North Macedonia); GRE2014/08 (Greece); BTV4-HUN2014 (Hungary); KOS2014/01 (Kosovo); and BTV-4/16-03 and BTV-4/17-15 (France)), indicating that the circulating strain in Kosovo is a re-emergent BTV-4 strain rather than a novel reassortant. BTV isolates sequenced in this study were assigned a genotype based on phylogenetic analysis of Segment 2 (Figure 2). All three samples from Kosovo clustered with other BTV-4 strains and were assigned to genogroup A based on segment 2 phylogeny (Figure 2). Both of the 2020 isolates from Kosovo formed a tight clade with other BTV-4 isolates from the region through the years 2014 to 2020, including those from North Macedonia (MKD2020/06), Hungary (HUN2014), and France (BTV-4/16-03 and BTV-4/17-15) (Figure 2). This clustering was also seen in segment 6 (encoding VP5) sequences (Figure 2). The results of percent nucleotide sequence (nt%) identity and phylogenetic analysis suggest that North Macedonia is a possible route of the incursion of BTV-4 into Kosovo. However, the lack of full genome sequences and detailed outbreak data available from other neighbouring countries and the Balkan region obscures the exact route.

## 4. Discussion

In this article, we report on the re-emergence of BTV-4 in Kosovo during 2020 and obtained the full genome sequence of the strain responsible for the outbreak.

**Figure 3:**
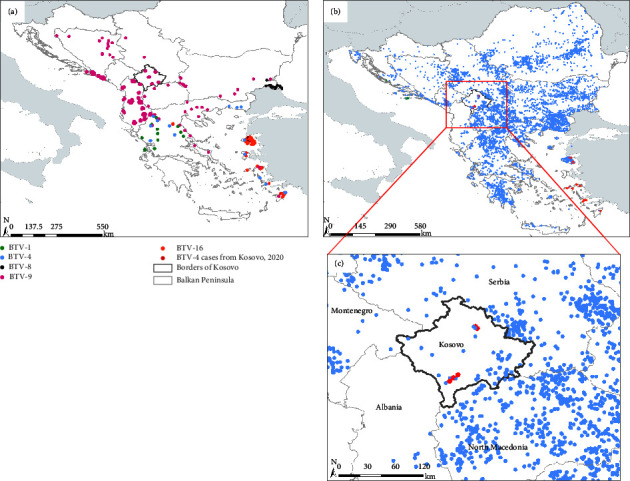
Maps of countries in the Balkan Peninsula showing the spatial distribution of BTV activity between 1998 and 2013 (a) and 2014 and 2020 (b), and closeup of Kosovo (c) to provide additional details in the region. White areas indicate Balkan countries, whereas countries outside of the Balkan Peninsula are shown by grey areas. BTV-1, BTV-4, BTV-8, BTV-9, and BTV-16 positive cases are represented by green, blue, black, purple, and orange dotted circles, respectively. Red dotted circles represent BTV-4 positive cases from the outbreak in Kosovo in 2020. All data of BTV occurrences were retrieved from the literature and the World Organisation for Animal Health (WOAH) database.

The distribution of BTV has changed dramatically in Europe during the last 25 years. In the Balkans, the breaking point for emergence of bluetongue was the BTV-9 outbreak on the Greek islands of Rhodes, Leros, Kos, and Samos in 1998 [20] (Figure 3(a)). Since then, BTV has extended over the Mediterranean basin and become well established in the south of the Balkan Peninsula. Following the 1998 outbreak in Greece, BTV-9 emerged in Bulgaria in 1999 apparently originating from neighbouring Turkey [21], before becoming widespread throughout the Balkan Peninsula by 2002. Kosovo reported its first clinical cases of bluetongue among sheep across seven municipalities in August 2001, caused by a BTV-9 strain also seen in an epizootic affecting neighbouring countries [15]. Over a decade later, BTV-4 emerged in Greece at the end of May 2014, with Bulgaria subsequently reporting cases in July 2014 [22] (Figure 3(b)). During the following months (August–December 2014), BTV-4 was reported in several Balkan countries (Albania, Croatia, Kosovo, North Macedonia, Montenegro, Romania, and Serbia). These outbreaks were mostly considered resolved by the following year, although there were further reports over the following years in some Balkan countries; Serbia and Montenegro, having the most recent cases to be resolved in October 2017 and January 2018, respectively. During both the initial and subsequent outbreaks, control measures varied by country with only a limited number of countries utilising vaccination campaigns [23]. Following years of apparent absence from the region, BTV-4 once again emerged in the Balkan Peninsula in July, 2020, when cases were confirmed in sheep and goats in North Macedonia [16]. The samples analysed in this study were collected at the start of September 2020, making Kosovo the second country in the region to be affected by the re-emerging strain (Figure 3(c)). In October, 2020, Bulgaria and Serbia also reported BTV-4 for the first time since 2015 and 2017, respectively [24]. Indeed, the epizootic followed a similar course to that of 2014, with cases reported from most countries in the Balkan Peninsula within the following months.

The first strain to be fully sequenced during the 2014 epidemic (HUN2014) revealed that it was a reassortant of BTV-1, BTV-2, BTV-4, and BTV-24 strains from African and Mediterranean countries [25]. However, since its initial characterisation, this strain has displayed a high degree of stability, with little variation at the nucleotide level over several years and circulation in numerous countries. This is displayed by the clustering of the strains from Balkan countries from both the 2014 and 2020 epizootics in the phylogenetic analysis of segment 2 sequences (the most variable segment of the BTV genome) (Figure 2). The samples collected in Kosovo in September 2020 and sequenced in the present study were most closely related to the strain seen at a similar time in North Macedonia (MKD2020/06), according to both nucleotide identity and phylogeny. Other closely related strains include those observed during the BTV-4 epizootic in the Balkans in 2014 (GRE2014/08 and HUN2014) and those seen in France (BTV-4/16-03 and BTV-4/17-15). The close relationship to the North Macedonian strain across all ten segments indicates that North Macedonia was a possible source of the BTV-4 incursion into Kosovo. However, cases were also recorded in other countries surrounding Kosovo at a similar time; therefore, these cannot be ruled out as the source of incursion, through either trade of infected animals or the movement of BTV-infected *Culicoides* midges across geographical borders. Kosovo is landlocked, sharing borders with four other countries, and as several *Culicoides* species capable of transmitting BTV are present throughout the Balkan region [26], there are plenty of opportunities for the spread of infected *Culicoides* into the country. The lack of available sequences from surrounding countries at a similar timepoint makes it difficult to determine the exact route of BTV into the country, highlighting the importance of generating full genome sequences of circulating BTV strains in their role in determining incursion routes.

An earlier study observed the presence of anti-BTV antibodies in the young livestock population of a limited number of municipalities in Kosovo in the years after an outbreak, despite no reports of clinical BT cases, indicating a subclinical circulation of the virus, albeit at low levels [15]. Another study found BTV seropositivity rates of 11.6% overall in municipalities across Kosovo in 2014-2015 [27]. Such widespread infection may have led to levels of natural immunity which decreased the prevalence of clinical disease, despite the absence of vaccinations. This subclinical circulation presents another possible route of emergence, especially when combined with an under-reporting of clinical cases as was seen in Greece in 2014 [23]. The regularity of outbreaks in Balkan countries, the serological evidence of subclinical circulation, and the stability of the causative strain may all suggest that BTV is now an endemic in the Balkan region, successfully overwintering each year.

The economic costs associated with BT outbreaks can be high through factors such as the loss of production, vaccination costs, and trade restriction measures [28]. The BTV-8 epidemic from 2006–2018 in Germany was estimated to cost between 157 and 203 million Euros [29]. Alongside the economic implications, BT is a disease of significant veterinary health importance due to the rapid spread and morbidity and case fatality rates. In 2021, the cattle, sheep, and goat populations were approximately 260,000, 211,000, and 30,000, respectively [30], representing a significant number of either susceptible or host animals. Taking into consideration that over 94% of farms in Kosovo are less than five hectares and are oriented towards subsistence farming [31], the impact of a BTV outbreak transcends the impact on the health and welfare of livestock, also representing a danger to food security in the region.

Under the framework of the Kosovo Food and Veterinary Agency, there is an administrative instruction MA-NO.28/2006 for controlling and eradicating BT disease. The measures prescribed by this legislation set in place specific provisions for the control and eradication of BT in the country, including quarantine, zoning, and restrictions on movements of certain animals of susceptible species in relation to BT [32]. However, despite vaccination being an efficient control strategy, BT vaccines were not used in Kosovo during or after either of the previous two outbreaks (2001 and 2014), and the country still lacks a vaccination program. The limited interventions to counteract the disease in Kosovo alongside circulation of two BTV serotypes (BTV-4 and BTV-9) over the past two decades in Kosovo emphasises the necessity for BTV surveillance and control measures. This not only needs to take place at a national level but also through multinational scientific cooperation, livestock disease management, and control strategies across the entire Balkan region to limit and prevent future epizootics.

## Figures and Tables

**Table 1:** The percent nucleotide sequence (nt%) identity for individual segments of BTV-KOS2020/01 and BTV-KOS2020/02 against closely related sequences from GenBank and a newly sequenced isolate from the 2014 BTV-4 outbreak in Kosovo (KOS2014/01).

Segment	Length (bp)	GenBank accession	Strain ID	Country, isolation date	KOS2020/01 nt% identity	KOS2020/02 nt% identity
1	3944	MT879201	MKD2020/06	North Macedonia, 2020	99.95	99.95
		MG944817	BTV-4/17-15	France, 2017	99.77	99.77
		MT879211	GRE2014/08	Greece, 2014	99.72	99.72
		KP268814	HUN2014	Hungary, 2014	99.67	99.67
		OP186416	KOS2014/01	Kosovo, 2014	99.62	99.67

2	2926	MT879202	MKD2020/06	North Macedonia, 2020	99.93	99.97
		MG944818	BTV-4/17-15	France, 2017	99.76^*∗*^	99.79^*∗*^
		MT879212	GRE2014/08	Greece, 2014	99.73	99.76
		KP268815	HUN2014	Hungary, 2014	99.66	99.69
		OP186417	KOS2014/01	Kosovo, 2014	99.72	99.76

3	2772	MT879203	MKD2020/06	North Macedonia, 2020	99.96	100
		MG944819	BTV-4/17-15	France, 2017	99.86^*∗*^	99.89^*∗*^
		MT879213	GRE2014/08	Greece, 2014	99.82	99.86
		KP268816	HUN2014	Hungary, 2014	99.78	99.82
		OP186418	KOS2014/01	Kosovo, 2014	99.82	99.86

4	1981	MT879204	MKD2020/06	North Macedonia, 2020	99.95	100
		MG944820	BTV-4/17-15	France, 2017	99.80^*∗*^	99.85^*∗*^
		MT879214	GRE2014/08	Greece, 2014	99.85	99.90
		KP268817	HUN2014	Hungary, 2014	99.85	99.90
		OP186419	KOS2014/01	Kosovo, 2014	99.85	99.90

5	1774	MT879205	MKD2020/06	North Macedonia, 2020	100	100
		MG944821	BTV-4/17-15	France, 2017	99.83	99.83
		MT879215	GRE2014/08	Greece, 2014	99.94	99.94^*∗*^
		KP268818	HUN2014	Hungary, 2014	99.89	99.89
		OP186420	KOS2014/01	Kosovo, 2014	99.89^*∗*^	99.77^*∗*^

6	1637	MT879206	MKD2020/06	North Macedonia, 2020	99.94	100
		MG944822	BTV-4/17-15	France, 2017	99.50^*∗*^	99.57^*∗*^
		MT879216	GRE2014/08	Greece, 2014	99.33	99.39
		KP268819	HUN2014	Hungary, 2014	99.33	99.39
		OP186421	KOS2014/01	Kosovo, 2014	99.45	99.51

7	1156	MT879207	MKD2020/06	North Macedonia, 2020	99.91	100
		MG944823	BTV-4/17-15	France, 2017	99.74	99.83
		MT879217	GRE2014/08	Greece, 2014	99.83^*∗*^	99.91^*∗*^
		KP268820	HUN2014	Hungary, 2014	99.91	100
		OP186422	KOS2014/01	Kosovo, 2014	99.91	100

8	1125	MT879208	MKD2020/06	North Macedonia, 2020	99.91	100
		MG944824	BTV-4/17-15	France, 2017	99.63^*∗*^	99.73^*∗*^
		MT879218	GRE2014/08	Greece, 2014	99.64	99.73
		KP268821	HUN2014	Hungary, 2014	99.64	99.73
		OP186423	KOS2014/01	Kosovo, 2014	99.64	99.73

9	1049	MT879209	MKD2020/06	North Macedonia, 2020	99.90	100
		MG944825	BTV-4/17-15	France, 2017	99.60^*∗*^	99.70^*∗*^
		MT879219	GRE2014/08	Greece, 2014	99.52	99.62
		KP268822	HUN2014	Hungary, 2014	99.52	99.62
		OP186424	KOS2014/01	Kosovo, 2014	99.62	99.71

10	822	MT879210	MKD2020/06	North Macedonia, 2020	100	100
		MG944826	BTV-4/17-15	France, 2017	99.88	99.88
		MT879220	GRE2014/08	Greece, 2014	99.51	99.51
		KP268823	HUN2014	Hungary, 2014	99.64	99.64
		OP186425	KOS2014/01	Kosovo, 2014	99.51	99.51

^*∗*^indicates <100% query coverage.

## Data Availability

The data that support the findings of this study are openly available in GenBank at https://www.ncbi.nlm.nih.gov/genbank/, reference numbers OP186396-OP186405 (KOS2020/01 segments 1-10), OP186406-OP186415 (KOS2020/02 segments 1-10), and OP186416-OP186425 (KOS2014/01 segments 1-10).
